# Accuracy and Reliability of Grip Strength Measurements: A Comparative Device Analysis

**DOI:** 10.3390/jfmk9040274

**Published:** 2024-12-16

**Authors:** Pascale Gränicher, Yael Maurer, Jörg Spörri, Bernhard Haller, Jaap Swanenburg, Rob A. de Bie, Ton A. F. Lenssen, Johannes Scherr

**Affiliations:** 1 University Center for Prevention and Sports Medicine, Balgrist University Hospital, University of Zurich, CH-8008 Zurich, Switzerland; yael.maurer@hotmail.com (Y.M.); joerg.spoerri@balgrist.ch (J.S.); johannes.scherr@balgrist.ch (J.S.); 2 Department of Epidemiology, CAPHRI School for Public Health and Primary Care, Maastricht University, 6200 MD Maastricht, The Netherlands; ra.debie@maastrichtuniversity.nl (R.A.d.B.); af.lenssen@mumc.nl (T.A.F.L.); 3 Department of Health Sciences and Technology, Institute of Human Movement Sciences and Sport, ETH Zurich, CH-8093 Zurich, Switzerland; 4 Sports Medical Research Group, Department of Orthopedics, Balgrist University Hospital, University of Zurich, CH-8008 Zurich, Switzerland; 5 Institute of AI and Informatics in Medicine, TUM School of Medicine and Health, Klinikum rechts der Isar, Technical University of Munich, 81675 Munich, Germany; bernhard.haller@tum.de; 6 Integrative Spinal Research ISR, Department of Chiropractic Medicine, Balgrist University Hospital, CH-8008 Zurich, Switzerland; jaap.swanenburg@balgrist.ch; 7 UZH Space Hub, Air Force Center, CH-8600 Dübendorf, Switzerland; 8 Institute of Anatomy, Faculty of Medicine, University of Zurich, CH-8057 Zurich, Switzerland; 9 Department of Physical Therapy, Maastricht University Medical Center MUMC+, 6229 HX Maastricht, The Netherlands

**Keywords:** hand strength, validation studies, reproducibility of results, test-retest reliability, physical therapy

## Abstract

**Background:** Grip strength is widely used as a surrogate parameter for functional status. The current gold standard, the JAMAR^®^ Hydraulic Dynamometer (JAMAR^®^ Hydraulic), presents challenges for individuals with painful finger joints or low grip strength. Therefore, the objective of this observational study was to assess comparability across the JAMAR^®^ Smart, the Martin Vigorimeter and the gold standard. Additionally, the intrasubject and intersession reliability of all three devices were examined, which are essential for quality control before generating normative data. **Methods:** Forty healthy participants (aged 37.0 ± 11.3 years) were included, and a subset of 20 participants was randomly selected for retesting. Grip strength testing involved five attempts per measurement device. **Results:** Intrasubject reliability was excellent (ICC3,1: 0.91–0.97), and a strong correlation (ICC3,1: 0.90–0.98) was found between the first attempt and the best of five as well as between the best of two or three attempts and the best of five, demonstrating robust reliability across multiple measures. Intersession reliability was good to excellent (ICC3,1: 0.85–0.97) across all three devices, which was confirmed by Bland–Altman analysis. The PCC (r) revealed moderate to very strong agreement with the current gold standard JAMAR^®^ Hydraulic (r = 0.68–0.98), with increased differences between the Martin Vigorimeter and the JAMAR^®^ Hydraulic measurements, particularly at low and high values. **Conclusions:** The proposed devices are suitable for test-retest analysis with 2–3 attempts. Despite high correlations between all three devices, the diverging levels of agreement between the Martin Vigorimeter and the established gold standard warrant caution against using these devices interchangeably.

## 1. Introduction

Maximum grip strength (MGS) serves as a cost-effective and easily accessible tool for assessing upper body strength across all age groups [1,2,3], with implications for overall body strength, lower limb strength and functional status [4,5,6,7]. Consequently, grip strength measurements are an effective method for monitoring progress in sports, rehabilitation and preventive care and guiding progression for training and therapy interventions [8,9,10]. Furthermore, a reduced MGS is correlated with increased mortality risk, frailty, sarcopenia and physical performance in older adults as well as diminished quality of life, underscoring its critical role in clinical and research contexts [11,12,13]. In younger individuals, decreased MGS is linked to impaired pulmonary capacity [14] and potentially poorer surgical outcomes [15].

Normative MGS data established by Mathiowetz et al. using the JAMAR^®^ Hydraulic Hand Dynamometer (JAMAR^®^ Hydraulic) for both children and adults [2,3,16] provide a benchmark for assessment. The JAMAR^®^ Hydraulic is regarded as the gold standard and is highly recommended by medical associations in both Europe (e.g., the European Society for Clinical and Economic Aspects of Osteoporosis, Osteoarthritis and Musculoskeletal Diseases (ESCEO)) and the USA (e.g., the American Society of Hand Therapists (ASHT)) as well as by the Collaborating Center for Public Health Aspects of Musculoskeletal Health and Aging of the World Health Organization (WHO) [16,17,18]. However, limitations such as weight, calibration needs and insensitivity in measuring low forces have led to consideration of alternative devices, such as the Martin Vigorimeter and the digital JAMAR^®^ Smart Hand Dynamometer (JAMAR^®^ Smart) [19]. Moreover, the strength values obtained with the JAMAR^®^ Hydraulic are frequently underestimated across various demographic groups, particularly among older individuals and those with weaker grips. Conditions such as painful arthritis of the hands and wrists or lack of motivation stemming from discomfort due to the rigid handle and isometric nature can significantly impact the validity of MGS measurements [20,21]. The pneumatic Martin Vigorimeter offers potential advantages over the current gold standard, particularly in providing a more comfortable grip through squeezing a pear-shaped rubber ball [22,23,24,25]. Similarly, in the context of children, it has been observed that while the firm grip required by the JAMAR^®^ Hydraulic does not inherently pose challenges, the 2 kg measurement increments may lack precision, especially for those with lower grip strength. Consequently, a digital model such as the JAMAR^®^ Smart, which incorporates electronic load cells for higher accuracy, may be preferable to its hydraulic counterpart [19,21,26].

Should either the JAMAR^®^ Smart or the Martin Vigorimeter demonstrate comparable quality criteria (such as intersession reliability and concurrent validity) to the established gold standard of the JAMAR^®^ Hydraulic, while also exhibiting superior applicability due to the abovementioned limitations, these two instruments could emerge as the new gold standard with universal applicability across diverse target groups. Another question regarding measurement of the MGS is the optimal number of attempts required to ascertain the true maximum strength for monitoring purposes in both research and clinical settings, with a focus on efficiency. The literature lacks consensus on the requisite number of attempts needed to determine the highest value, as test protocols and data analysis methodologies vary considerably (e.g., utilization of the best attempt versus averaging all attempts) [27]. Therefore, the objectives of this study are twofold: (1) determine the intrasubject and intersession reliability of the three commonly used grip strength assessment devices and (2) evaluate the concurrent validity of the JAMAR^®^ Smart and Martin Vigorimeter relative to the current gold standard of the JAMAR^®^ Hydraulic.

## 2. Materials and Methods

### 2.1. Experimental Approach to the Problem

This observational study with randomized sequences of repeated measurements was conducted at Balgrist University Hospital in Zurich, Switzerland. The study protocol was registered and previously published on https://clinicaltrials.gov/study/NCT05594888 (accessed on 13 December 2024), and it was approved by the local ethics committee of Zurich, Switzerland prior to recruitment (BASEC-Nr. 2022-01400). This study was conducted in accordance with the Good Clinical Practice guidelines, the guiding principles of the Declaration of Helsinki, and reported according to the Strengthening the Reporting of Observational Studies in Epidemiology (STROBE) statement [28].

### 2.2. Subjects

#### 2.2.1. Recruitment and Eligibility Criteria

Healthy male and female volunteers were consecutively recruited among the staff of the Balgrist University Hospital and Sportamt Stadt Zürich in Switzerland. Eligibility for study participation was assessed by the study team (P.G. and Y.M.). The inclusion criteria were adults (1) aged 18–64 years and (2) with a body mass index < 30 kg/m^2^. The exclusion criteria were (1) disease or injury (e.g., musculoskeletal or neurological) which could affect upper extremity strength; (2) physical impairment of the upper extremities (visible swelling or the presence of a splint or cast); (3) acute pain or infection; (4) cognitive impairment (inability to understand and follow directions); and (5) insufficient knowledge of the German language. By choosing a homogeneous cohort of healthy, working-age adults, we minimized the confounding variables related to age and BMI, which could otherwise affect the validity of the device comparisons. According to the comparison studies of Mathiowetz et al. and Amaral et al., 40 volunteers (20 females and 20 males) were recruited and enrolled in this study [29,30]. This sample size was considered sufficient because the validity and reliability of the 3 instruments had been tested previously, albeit not in this constellation [19,20,31,32,33]. All patients provided formal written informed consent before the test.

#### 2.2.2. Randomization

To randomize the sequences of the test devices (JAMAR^®^ Hydraulic, JAMAR^®^ Smart, and Martin Vigorimeter) and the starting hand (left or right), two dice were rolled. The first dice determined the device sequence as follows: 1 = Hydraulic—Smart—Martin, 2 = Smart—Martin—Hydraulic, 3 = Martin—Hydraulic—Smart, 4 = Smart—Hydraulic—Martin, 5 = Hydraulic—Martin—Smart, and 6 = Martin—Smart—Hydraulic. The second dice determined the starting hand (1–3 = left hand; 4–6 = right hand).

**Figure 1:**
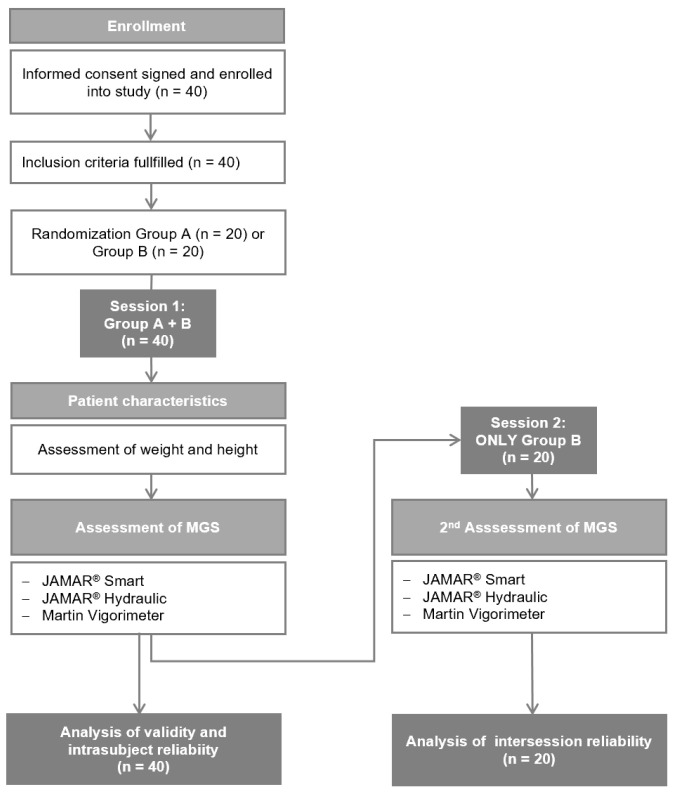
CONSORT flow diagram of the progression of study participants in groups A and B through the phases of the study. N = number of participants; MGS = maximum grip strength.

To allocate participants to groups for assessing intersession reliability, 40 participants were randomly assigned to Group A (one test session) or Group B (two test sessions) by blindly selecting slips of paper labeled “Group A” or “Group B” from an envelope. Twenty participants were allocated to Group B for the second measurement (Figure 1).

### 2.3. Procedures

**Figure 2:**
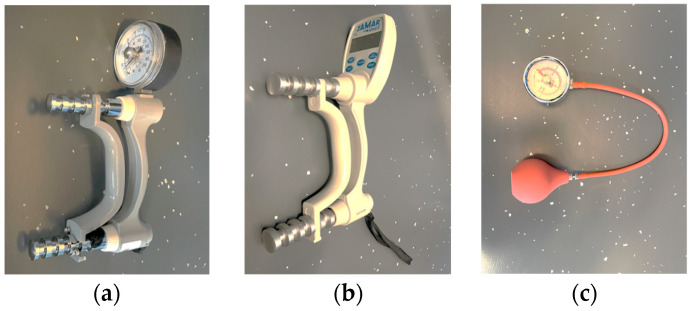
(**a**–**c**) JAMAR^®^ Hydraulic, JAMAR^®^ Smart, and Martin Vigorimeter.

The test-retest reliability and concurrent validity of the MGS measurements were assessed with a JAMAR^®^ Hydraulic (Figure 2a; Performance Health Supply Inc., Warrenville, IL, USA) compared with those of a JAMAR^®^ Smart (Figure 2b; Performance Health Supply Inc., Warrenville, IL, USA) and a Martin Vigorimeter (Figure 2c; Firma Gebrüder Martin, Tuttlingen, Germany).

For standardization, the adjustable handle of both JAMAR^®^ dynamometers was set to the second position for all participants [34]. Balloon number 5 of the Martin Vigorimeter was used due to its high correlation with the second handle position of the JAMAR^®^ dynamometers [34]. The procedures for body position and verbal instructions were standardized according to the ASHT [17]. An identical position was used for measurements conducted with the Martin Vigorimeter. For each device, the participants had one attempt with 50% MGS to become accustomed to handling it.

Forty participants underwent testing with each of the 3 devices, assessing the MGS for both the dominant and nondominant hands. Each participant completed 5 consecutive attempts with each device, with a 30 s rest period between attempts. The testing of 20 randomly selected participants (Group B) was subsequently repeated on a second day (session 2) to evaluate intersession reliability. The highest values recorded from the 5 attempts per device and hand were used for both validity assessment and intersession reliability analysis [8]. Furthermore, to examine intrasubject reliability, the results from all 5 attempts per hand and device in session 1 were analyzed.

### 2.4. Statistical Analyses

Descriptive statistics were used to describe the parametric data, and the participants’ characteristics are presented as the means ± standard deviations (SDs), numbers and frequencies. The normality of the data was assessed by examining histograms and the Kolmogorov–Smirnov test. Categorical data are presented as absolute and relative frequencies. The data were entered into an electronic database (REDCap^®^), and analyses were carried out using statistical software (SPSS version 29.0, Chicago, IL, USA) and Excel (Microsoft Office Professional Plus 2019). Two-sided tests were performed, and a significance level of α = 0.05 was used for each test.

#### 2.4.1. Reliability

The intraclass correlation coefficient (ICC3,1) [35] and 95% confidence interval (CI) were calculated using the spreadsheet provided by Hopkins [36] for consecutive pairwise measurements to express intrasubject and intersession reliability [37]. Reliability was rated as poor (<0.50), moderate (0.50–0.75), good (0.75–0.90) or excellent (>0.90) based on the classification proposed by Koo and Li [31]. To determine the standard error of measurement (SEM), the following formula was used [38]: $SEM=SD\;difference/\sqrt{2}$. The smallest detectable difference (SDD) was then calculated using the SEM [39,40], where $SDD=1.96\times \sqrt{2}\times SEM$.

The intrasubject reliability analysis included data from all 40 participants. Repeated measures analysis of variance (ANOVA) was conducted, and the within-subject factor ATTEMPT (attempts 1–5) was utilized. The intersession reliability was analyzed for 20 randomly selected participants (Group B). The ICC3,1, SEM and SDD were computed based on the best of 5 attempts per day and device. Bland–Altman analyses were performed to examine the agreement between session 1 and session 2, plotting the absolute and relative differences alongside the mean values of the sessions [41]. Additionally, 95% limits of agreement (LoAs) were calculated (mean difference ± 1.96 SD of differences) and visually inspected for indicators of good agreement, including MDs close to zero and a uniform distribution across the measurement range [41,42].

#### 2.4.2. Validity

Concurrent validity was evaluated using the Hopkins spreadsheet, which employs linear regression and calculates the Pearson correlation coefficient (PCC, r) with 95% CIs from the best of 5 attempts for each of the 3 devices for both hands. This approach aimed to quantify the relationship between measurements from the JAMAR^®^ Smart and Martin Vigorimeter and measurements based on the gold standard of the JAMAR^®^ Hydraulic [37]. The correlation magnitude was interpreted as follows: r < 0.3 is negligible; 0.3–0.5 is low; 0.5–0.7 is moderate; 0.7–0.9 is strong and >0.9 is very strong [43]. Additionally, the agreement of measurements to explore interchangeable utilization of the devices was assessed through Bland–Altman analyses [44].

## 3. Results

### 3.1. Participants

Between December 2022 and January 2023, 40 volunteers were enrolled in this study (20 in Group A and 20 in Group B) (Figure 1). The demographics and characteristics of the participants are presented in Table 1. No significant differences were observed between groups A and B.

### 3.2. Intrasubject Reliability

**Figure A1:**
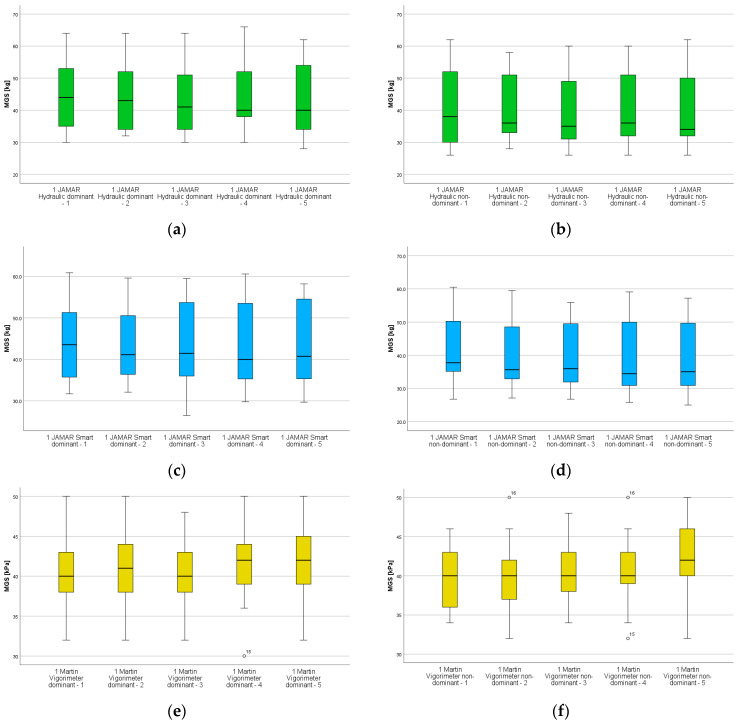
(**a**–**f**) Boxplots of attempts 1–5 for each device and both hands.

Within-subject variation of attempts 1–5 showed excellent correlation (ICC3,1 > 0.90) for all devices and for both hands (Appendix A, Table A1). To determine the ideal number of attempts for obtaining reliable results, the first attempt, the best of two attempts and the best of three attempts were compared with the best of five attempts (Table 2). The comparisons yielded excellent correlations (ICC3,1 > 0.9) across all three devices, regardless of hand dominance or the number of attempts made. An analysis of the best results out of five attempts for both the dominant and nondominant hands revealed no significant differences (two-sided *t*-test: *p* > 0.05) and correlations ranging from good (Martin Vigorimeter: ICC3,1 = 0.83, 95% CI (0.71–0.91)) to excellent (JAMAR^®^ Hydraulic: ICC3,1 0.95, 95% CI (0.92–0.98); JAMAR Smart^®^: ICC3,1 = 0.93, 95% CI (0.86–0.96)) (Table 3). Although significant differences in MGS were observed by the three devices, they seemed to measure within similar ranges overall across the five attempts (Table 4; Appendix A, Figure A1).

### 3.3. Intersession Reliability

The correlation between the two sessions ranged from good (ICC3,1 0.85, Martin Vigorimeter) to excellent (ICC3,1 ≥ 0.95, both JAMAR^®^ dynamometers) for all three devices irrespective of hand dominance, with relatively narrow CIs except for the Martin Vigorimeter (Table 5).

**Figure 3:**
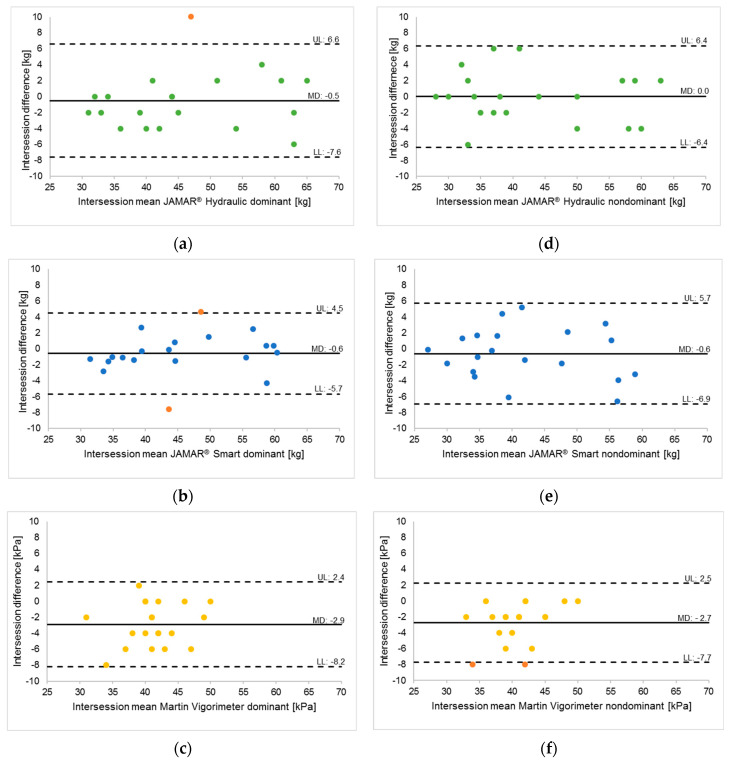
(**a**–**f**) Bland-Altman plots of differences between session 1 and session 2 for both dominant (**a**–**c**) and nondominant (**d**–**f**) hands for all 3 devices (*n* = 20). The limits of agreement are represented as dotted lines (from −1.96 to +1.96 SD). LL = lower limits of agreement; UL = upper limits of agreement. Mean differences (MDs) are shown as solid black lines. • = outliers. (**a**) JAMAR^®^ Hydraulic, dominant (green dots); (**b**) JAMAR^®^ Smart, dominant (blue dots); (**c**) Martin Vigorimeter, dominant (yellow dots); (**d**) JAMAR^®^ Hydraulic (green dots), nondominant; (**e**) JAMAR^®^ Smart, nondominant (blue dots) and (**f**) Martin Vigorimeter, nondominant (yellow dots).

MGS differences between sessions 1 and 2 were plotted against their means, and the LoAs are presented in the Bland–Altman plots in Figure 3 for both the dominant (a–c) and nondominant (d–f) hands and all three devices. Taking the MGS values assessed with the JAMAR^®^ Smart as an example, there was a 68% probability that the MGS values obtained from repeated measurements with the dominant hand would fall within approximately ± 1.8 kg.

### 3.4. Validity

**Figure 4:**
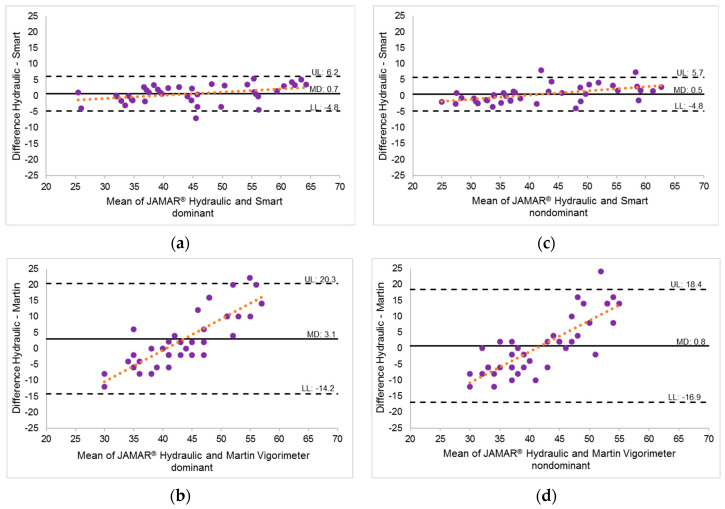
(**a**–**d**) Bland–Altman analyses of comparisons of the JAMAR^®^ Smart and Martin Vigorimeter to the gold standard JAMAR^®^ Hydraulic, assessing MGS in both the dominant (**a**,**b**) and nondominant (**c**,**d**) hands (*n* = 40). Mean differences (MDs) are shown as solid black lines, and the trendlines are presented as orange dotted lines. LL = lower limit of agreement; UL = upper limit of agreement (limits of agreement are represented as dashed black lines (from −1.96 to +1.96 SD)). (**a**) JAMAR^®^ Hydraulic vs. JAMAR^®^ Smart, dominant; (**b**) JAMAR^®^ Hydraulic vs. Martin Vigorimeter, dominant; (**c**) JAMAR^®^ Hydraulic vs. JAMAR^®^ Smart, nondominant and (**d**) JAMAR^®^ Hydraulic vs. Martin Vigorimeter, nondominant.

Concurrent validity analysis revealed a very strong relationship between the two JAMAR^®^ devices, with correlations of r = 0.97 (95% CI: 0.95–0.99) for the dominant hand and r = 0.98 (95% CI: 0.96–0.99) for the nondominant hand. Additionally, a moderate-to-strong relationship was shown between the Martin Vigorimeter and JAMAR^®^ Hydraulic, with r = 0.68 (95% CI: 0.47–0.82) for the nondominant hand and r = 0.72 (95% CI: 0.52–0.84) for the dominant hand. The agreement between the MGS measurements obtained with the JAMAR^®^ Smart or Martin Vigorimeter and those obtained with the JAMAR^®^ Hydraulic is illustrated in Figure 4a–d, which depicts the Bland–Altman analyses. Figure 4a,c illustrates the notable agreement between the JAMAR^®^ Smart and the gold standard of the JAMAR^®^ Hydraulic, with minimal MDs and LoAs alongside a well-distributed range of values across all strength levels. Conversely, the comparison depicted in Figure 4b,d, between the Martin Vigorimeter and the gold standard reveals a distinct trend: The measurements obtained from the Martin Vigorimeter tended to underestimate individuals with higher MGSs and, conversely, overestimate those with lower MGSs, which is evident from the observed slope of differences. The presence of considerable LoAs despite relatively low MDs underscores this disparity.

## 4. Discussion

This study’s findings support the use of the JAMAR^®^ Hydraulic, the JAMAR^®^ Smart and the Martin Vigorimeter for assessing MGS in healthy adults. Across all three devices, high ICCs over five attempts and across two test sessions were observed, indicating good-to-excellent intrasubject and intersession reliability. However, the validity assessment yielded fewer definitive results. Despite relatively high correlations between the MGS values obtained from the three devices, Bland–Altman analysis revealed considerable variations among the participants across the measurement range when comparing the Martin Vigorimeter with the JAMAR^®^ Hydraulic device.

### 4.1. Intrasubject Reliability

To optimize the efficiency of MGS measurements without compromising accuracy, the optimal number of attempts needed was explored. Despite individual differences, excellent correlations among the five attempts were found (ICC3,1 > 0.9), irrespective of device or hand dominance. Comparing the best of five attempts with fewer attempts showed only a negligible decrease in the ICC, suggesting that 1–3 attempts may suffice to obtain reliable MGS values. Moreover, Coldham et al. [45] argued that a single attempt is less fatiguing than three attempts but equally reliable, a notion expressed in Mathiowetz’s findings, which revealed no fatigue effect when comparing 1–3 attempts [46]. These observations align with the conclusions drawn by Reijnierse et al. [47], who analyzed three consecutive attempts with the JAMAR^®^ Hydraulic across various populations, and Hamilton et al. [48], who reported similar test-retest reliability for a single attempt, the mean of two or three attempts and the best of three attempts using the JAMAR^®^ dynamometer. However, it is worth noting that Reijnierse et al. [47] cautioned against potential underestimation of the MGS with fewer than three attempts, as even a 1 kg difference in grip strength has been associated with a greater risk of mortality among elderly individuals, according to a meta-analysis by Cooper et al. [49]. While conducting fewer attempts can save time, it is crucial to consider potential accuracy implications, especially in populations where grip strength is critical for health outcomes. Given that the dominant hand tended to yield slightly higher values, assessing only the dominant hand may suffice to ascertain the maximum strength values, as confirmed by Stamate et al. [50].

### 4.2. Intersession Reliability

This study demonstrated high reproducibility for MGS values across both test occasions using all three devices irrespective of hand dominance and without any systematic changes. Coupled with minimal measurement errors, these findings suggest that these devices offer consistent results with repeated measurements, enabling the detection of small yet clinically relevant changes in MGS measurements. Similar findings were reported by Sipers et al. [20], who found no significant difference between two sessions in their study with geriatric patients.

Negative MDs and smaller MGS values in the second test session suggest the exclusion of a learning effect but possibly indicate a lack of motivation. Furthermore, we found no apparent association between the difference and the mean value of either session, suggesting that the measurements during both sessions were reliable across the entire range of possible values. Therefore, clinicians and coaches can confidently interpret an increase of more than 5.3 kPa measured with the Martin Vigorimeter from one day to the next as reflecting a true change in the patient’s or athlete’s MGS and not a fluctuation within the device.

### 4.3. Validity

The correlation between the MGS values measured with the Martin Vigorimeter and the JAMAR^®^ Smart, compared with those obtained with the JAMAR^®^ Hydraulic, ranged from moderate (r = 0.68) to very strong (r = 0.98), which is consistent with previous research [24,51]. However, substantial discrepancies were found between the MGS values obtained with the Martin Vigorimeter and those of the gold standard. The SEMs indicated a considerable lack of agreement and proportional difference variability among the participants, particularly at extreme MGS values. The Bland–Altman plots confirmed these findings, and while the MDs were within acceptable limits, notable individual differences were evident. Specifically, the observed slope in the plots indicated a trend where the Martin Vigorimeter tended to register higher strength values in individuals with lower strength levels, gradually transitioning to lower values compared with the gold standard of the JAMAR^®^ Hydraulic in individuals with higher strength levels.

One explanation for these discrepancies lies in the distinct biomechanics between the grips on the JAMAR^®^ devices and the pneumatic Martin Vigorimeter [33,51]. Gripping the JAMAR^®^ device induces isometric contraction and a lack of feedback due to the static nature of the grip. Conversely, the Martin Vigorimeter elicits a concentric contraction, offering both visual and haptic feedback through rubber bulb squeezing and potentially resulting in higher scores, particularly among weaker individuals. Additionally, participants with lower strength values tended to score lower with the JAMAR^®^ Hydraulic device than the Martin Vigorimeter device, possibly due to discomfort during device handling [19].

Therefore, due to the widening trend of the agreement range with lower or higher strength values, we advise not using the Martin Vigorimeter interchangeably with the JAMAR^®^ hydraulic gold standard for clinical practice or research purposes. Despite the large measurement errors associated with the Martin Vigorimeter, the high PCC measured for the dominant hand suggests good validity at the population level, indicating that the device may still be suitable for monitoring discrimination within the same person. These findings underscore the importance of careful consideration when selecting and interpreting MGS measurements with different devices in clinical, training and research settings. However, if the assessment were conducted in the context of hand rehabilitation, where injuries or pathologies could affect or limit hand closure, the devices would likely evaluate different constructs due to their varying mechanical demands.

### 4.4. Strengths and Limitations

#### 4.4.1. Strengths

To mitigate protocol-related errors, a standardized testing procedure was rigorously followed, including maintaining a consistent posture and providing standardized instructions and encouragement to participants. To minimize familiarization or learning bias, a practice attempt with 50% of the maximum force was conducted with each device [35]. Moreover, multiple measures of reliability were provided, and the confidence intervals were calculated as recommended by Hopkins et al. [35] to ensure meaningful reporting. No cases of drop-out or loss to follow-up were recorded, indicating high adherence to the protocol and reflecting feasibility in clinical and training practice.

#### 4.4.2. Limitations

This study’s generalizability is limited by the fact that all participants were aged 18–59 years, which may not accurately represent populations in other age groups. However, targeting healthy adults allowed us to establish a baseline of device validity and reliability where potential variables affecting the accuracy of MGS measurements were minimized. The main focus of this study was to assess the agreement of measurement of alternative devices against the gold standard rather than to specifically examine the MGS across a variety of populations. Therefore, a homogeneous cohort reduced potential bias and ensured that the differences in device readings were due to the instruments themselves rather than variability in factors such as age, BMI or underlying health conditions. To assess whether the reliability observed in this study remains consistent in populations with specific pathologies, such as neuromuscular disorders or hand injuries, separate studies need to be conducted. This study presents a first step in laying the groundwork for future research in clinical populations and across age groups.

Additionally, the sample size, which is common in reliability studies with continuous data, could affect the precision of the estimated LoA and limit the generation of clinically useful SDDs [39,52]. While the sample size was adequate for assessing device reliability, a larger sample might be necessary to generate SDDs which are generalizable for monitoring meaningful changes over time. Moreover, the high testing volume with five attempts and three devices might have influenced the results, with the sequence of devices potentially affecting outcomes, as demonstrated by Statmate et al. [50].

Our results serve as a crucial quality control measure by establishing intrasubject and intersession reliability as well as concurrent validity within the test protocol. These aspects are vital for ensuring accuracy and consistency in subsequent efforts to generate normative data for the three devices, accounting for factors such as age, sex, physical activity level and underlying medical conditions. To generate reference values, we recommend conducting 2–3 attempts with each hand and all three devices.

## 5. Conclusions

The JAMAR^®^ Smart device has emerged as a valid and reliable alternative to the current gold standard for assessing MGS, offering more precise measurement intervals. We recommend its utilization across diverse populations in physiotherapeutic clinical, training and research settings. Based on the current results, in cases of low tolerance, such as painful finger joints, the Martin Vigorimeter is preferable. Assessing the dominant hand alone is suitable as a surrogate parameter, particularly for evaluating lower extremity strength (e.g., monitoring, intervention evaluation and independence goal setting). However, in hand rehabilitation or explicit hand grip strength training (e.g., climbing), where comparison with the healthy or stronger side is essential, assessment of both hands is advised. All three devices are reliable options for assessing grip strength, and the JAMAR^®^ Hydraulic may be replaced by the JAMAR^®^ Smart. The Martin Vigorimeter, however, is not advisable for interchangeable use due to its low agreement of measurement and distinct pattern across attempts and grip strength ranges.

## Figures and Tables

**Table 1:** Demographic information and characteristics of the study cohort.

	Total (n = 40)		Group A (n = 20)		Group B (n = 20)		*p* Value (Group)
	Mean	(SD)	Mean	(SD)	Mean	(SD)	
Sex (f:m)	20:20		10:10		10:10		1.00
Age (years)	37.0	(11.3)	36.4	(11.6)	37.6	(11.2)	0.74
Height (cm)	173.9	(10.0)	175.3	(11.1)	172.6	(8.8)	0.40
Weight (kg)	72.4	(14.0)	73.4	(15.2)	71.4	(13.1)	0.65
BMI (kg/m^2^)	23.7	(2.7)	23.7	(2.8)	23.8	(2.8)	0.89
Handedness (r:l)	35:5		18:2		17:3		0.64

f = female; l = left-handed; m = male; n = number of participants; *p* = independent *t*-test; r = right-handed; SD = standard deviation.

**Table 2:** Comparison between the first attempt, the best of two attempts and the best of three attempts and the best of five attempts (N = 40).

		First vs. Best of 5			Best of 2 vs. Best of 5			Best of 3 vs. Best of 5		
Device	Hand	ICC3,1	(95% CI)	SDD	ICC3,1	(95% CI)	SDD	ICC3,1	(95% CI)	SDD
JAMAR^®^ Hydraulic (kg)	dom.	0.98	(0.97–0.99)	4.2	0.99	(0.98–0.996)	3.0	0.996	(0.99–0.998)	2.2
	nondom.	0.96	(0.92–0.98)	7.0	0.98	(0.97–0.99)	4.2	0.995	(0.99–0.997)	2.3
JAMAR^®^ Smart (kg)	dom.	0.94	(0.88–0.97)	7.1	0.95	(0.90–0.97)	6.5	0.99	(0.99–0.996)	2.4
	nondom.	0.99	(0.98–0.995)	2.8	0.996	(0.99–0.998)	1.7	0.99	(0.996–0.999)	1.4
Martin Vigorimeter (kPa)	dom.	0.90	(0.82–0.95)	3.9	0.95	(0.91–0.97)	2.8	0.96	(0.93–0.98)	2.5
	nondom.	0.91	(0.84–0.95)	3.7	0.94	(0.88–0.97)	3.2	0.96	(0.93–0.98)	2.5

CI = confidence interval; dom. = dominant hand; ICC = intraclass correlation coefficient; N = number of participants; nondom. = nondominant hand; SDD = smallest detectable difference.

**Table 3:** Correlations between the best of 5 attempts of the dominant and nondominant hands.

Device		N	Mean	SD	*p*	ICC3,1	95% CI	SEM	SDD
JAMAR^®^ Hydraulic (kg)	dom.	40	44.9	11.3	0.25	0.95	(0.92–0.98)	2.5	7.0
	nondom.	40	45.3	11.5					
JAMAR^®^ Smart (kg)	dom.	40	44.1	9.9	0.23	0.93	(0.86–0.96)	2.9	8.0
	nondom.	40	44.6	10.4					
Martin Vigorimeter (kPa)	dom.	40	41.2	4.3	0.5	0.83	(0.71–0.91)	1.9	5.3
	nondom.	40	42.2	4.5					

CI = confidence interval; dom. = dominant hand; ICC = intraclass correlation coefficient; N = number of participants; *p* = two-sided *t*-test; nondom. = nondominant hand; SD = standard deviation; SDD = smallest detectable difference; SEM = standard error of measurement.

**Table 4:** Intrasubject reliability was analyzed using attempts 1–5 per hand and device from 40 participants.

		JAMAR^®^ Hydraulic (kg)					JAMAR^®^ Smart (kg)					Martin Vigorimeter (kPa)				
Hand	Attempt	Mean	SD	Min	Max	Main Effect *p*	Mean	SD	Min	Max	Main Effect *p*	Mean	SD	Min	Max	Main Effect *p*
dom.	1	44.0	11.8	20.0	66.0	<0.001 *	42.6	9.5	24.9	60.9	0.51	39.9	4.3	32.0	50.0	<0.001 *
	2	43.1	11.0	24.0	64.0		41.8	9.6	25.0	59.6		40.5	4.4	32.0	50.0	
	3	42.0	10.6	24.0	64.0		42.0	10.3	24.5	59.5		40.4	4.0	32.0	50.0	
	4	42.2	11.2	20.0	66.0		41.8	10.5	23.8	60.6		41.0	4.5	30.0	50.0	
	5	42.0	11.2	16.0	62.0		41.9	10.2	21.4	62.5		41.3	4.4	32.0	50.0	
nondom.	1	40.0	11.7	22.0	64.0	0.23	40.7	10.0	24.8	61.3	<0.001 *	39.4	4.1	32.0	50.0	<0.001 *
	2	39.9	10.7	22.0	62.0		39.2	10.7	22.2	59.8		39.4	4.7	30.0	50.0	
	3	39.0	11.3	20.0	60.0		38.8	10.2	19.9	57.1		40.0	4.5	30.0	50.0	
	4	39.1	10.4	24.0	60.0		38.3	10.5	21.1	60.6		40.1	4.7	32.0	52.0	
	5	38.8	10.7	20.0	62.0		38.3	10.2	23.3	57.2		41.0	5.1	32.0	52.0	

* Significant at *p* < 0.05. CI = confidence interval; dom. = dominant hand; Max = maximum; Min = minimum; nondom. = nondominant hand; SD = standard deviation.

**Table 5:** Absolute and relative reliability scores of MGS measurements for the best of five attempts in sessions 1 and 2 for all devices and both hands (N = 20).

Device	Hand	No.	Mean	SD	ICC3,1 (95% CI)	SEM	SDD
JAMAR^®^ Hydraulic (kg)	dom.	1	46.4	11.0	0.95 (0.89–0.98)	2.5	7.1
		2	45.9	11.6			
	nondom.	1	42.9	11.6	0.96 (0.91–0.99)	2.3	6.3
		2	42.9	11.3			
JAMAR^®^ Smart (kg)	dom.	1	45.9	9.7	0.97 (0.92–0.99)	1.8	5.1
		2	45.3	10.1			
	nondom.	1	42.3	10.3	0.95 (0.89–0.98)	2.3	6.3
		2	41.7	9.7			
Martin Vigorimeter (kPa)	dom.	1	42.8	4.5	0.85 (0.67–0.94)	1.9	5.3
		2	39.9	5.0			
	nondom.	1	42.1	4.0	0.85 (0.66–0.94)	1.8	5.0
		2	39.4	4.8			

No. = session number; dom. = dominant hand; ICC = intraclass correlation; N = number of participants; nondom. = nondominant hand; SD = standard deviation; SDD = smallest detectable difference; SEM = standard error of measurement.

## Data Availability

The datasets generated and analyzed during the current study are not publicly available but are available from the corresponding author (P.G.) upon reasonable request.

## Appendix A

**Table A1:** Correlations between all 5 attempts per hand and device.

Device	Hand	N	Mean	SD	ICC_3,1_ (95% CI)	SEM	SDD
JAMAR^®^ Hydraulic (kg)	dom.	40	42.7	11.2	0.96 (0.94–0.98)	2.3	6.3
	nondom.	40	39.3	11.0	0.95 (0.93–0.97)	2.4	6.8
JAMAR^®^ Smart (kg)	dom.	40	42.0	10.0	0.96 (0.94–0.98)	2.0	5.5
	nondom.	40	39.0	10.3	0.96 (0.94–0.98)	2.0	5.6
Martin Vigorimeter (kPa)	dom.	40	40.6	4.3	0.91 (0.85–0.94)	1.4	3.8
	nondom.	40	40.0	4.6	0.92 (0.88–0.95)	1.3	3.7

CI = confidence interval; dom. = dominant hand; ICC = intraclass correlation coefficient; N = number of participants; nondom. = nondominant hand; SD = standard deviation; SDD = smallest detectable difference; SEM = standard error of measurement.
